# Interaction of Erdosteine with TrkA Signaling Pathways: Implications for Analgesia

**DOI:** 10.3390/ijms26094079

**Published:** 2025-04-25

**Authors:** Nicoletta Marchesi, Stefano Govoni, Clive P. Page, Luda Diatchenko, Alessia Pascale, Piercarlo Fantucci, Jacopo Vertemara, Silvia Natoli, Massimo Allegri

**Affiliations:** 1 Department of Drug Sciences, Pharmacology Section, University of Pavia, 27100 Pavia, Italy; nicoletta.marchesi@unipv.it (N.M.); govonis@unipv.it (S.G.); 2 Institute of Pharmaceutical Science, King’s College London, London SE1 9NH, UK; clive.page@kcl.ac.uk; 3 Faculty of Dental Medicine and Oral Health Sciences, Department of Anesthesia, Faculty of Medicine, Alan Edwards Centre for Research on Pain, McGill University, Montreal, QC H3A 1G1, Canada; luda.diatchenko@mcgill.ca; 4 Department of Biotechnology and Biosciences, University of Milan-Bicocca, 20126 Milan, Italy; piercarlo.fantucci@unimib.it (P.F.); jacopo.vertemara@unimib.it (J.V.); 5 Department of Clinical-Surgical, Diagnostic and Pediatric Sciences, University of Pavia, 27100 Pavia, Italy; silvia.natoli@unipv.it; 6 IRCCS Policlinco San Matteo, 27100 Pavia, Italy; 7 Centre Lémanique de Neuromodulation et Thérapie de la Douleur, Ensemble Hospitalier de la Côte (EHC), 1110 Morges, Switzerland; massimo.allegri@ehc.vd.ch

**Keywords:** pain, non-opioid drugs, NGF, TrkA, drug repurposing

## Abstract

Thiol-containing drugs may interact with a region of tropomyosin receptor kinase A (TrkA), potentially inhibiting its activation by nerve growth factor (NGF). This action has been linked to potential analgesic activities. Here, we describe the ability of erdosteine, a thiolic compound classified as a mucolytic agent, to bind to the TrkA receptor sequence in silico and its in vitro effects on TrkA activation induced by NGF in cultured human neuroblastoma cells. Our results show that erdosteine and its metabolite, Met-1, bind to the TrkA receptor pocket, involving the primary TrkA residues Glu331, Arg347, His298, and His297. Furthermore, Met-1 has the ability to reduce the disulfide bridge between Cys300 and Cys345 of TrkA. In vitro measurement of TrkA autophosphorylation following NGF activation confirmed that erdosteine and Met-1 interfere with NGF-induced TrkA activation, leading to a consequent loss of the molecular recognition and spatial reorganization necessary for the induction of the autophosphorylation process. This effect was inhibited by low millimolar concentrations of the two compounds, reaching a maximal inhibition (around 40%) after 24 h of exposure to 1 mM erdosteine, and then plateauing. These findings suggest that erdosteine can act as a TrkA antagonist, thus indicating that this drug may have potential as an analgesic via a novel non-opioid mechanism of action operating through NGF signaling inhibition at the level of TrkA.

## 1. Introduction

Thiol compounds, such as erdosteine, a drug classified as a mucolytic agent acting via redox mechanisms and primarily used in the treatment of respiratory diseases such as chronic obstructive pulmonary disease (COPD), also possesses anti-inflammatory properties [1,2]. Thus, it is plausible that these pharmacological properties of thiols may be useful in the treatment of other disorders beyond their use in pulmonary diseases, particularly where oxidative stress and inflammation are involved in the pathogenesis of acute and chronic pain syndromes. Moreover, literature data support the concept that some thiol compounds may act on nerve growth factor (NGF) signaling, a pathway linked to nociception and nociplastic pain [3,4,5].

There is now considerable interest in further understanding whether thiol drugs, such as erdosteine, could be repurposed as treatments for pain. Indeed, Cazzola and co-workers [1,2] showed that erdosteine and thiol-based agents may reduce oxidative stress directly through the presence of free sulfhydryl groups (as in Met-1, the primary active metabolite of erdosteine), serving as a source of reducing equivalents, and indirectly through intracellular glutathione (GSH) replenishment. Moreover, thiol compounds, including erdosteine, may elicit anti-inflammatory effects through NF-κB activation and a subsequent reduction in IL-6 and IL-1β release [1,2]. Furthermore, the modulation of oxidative processes in the spinal cord could help to relieve or prevent chronic pain [6].

Multiple lines of evidence indicate a significant role for NGF and its TrkA receptor signaling pathway in mediating diverse biological activities. These activities may be characterized as either advantageous, such as those supporting neuronal survival, or disadvantageous, as exemplified by their involvement in osteoarthritic, low-back, and neuropathic pain conditions [7]. It has also been found that mutations in the *NGF* gene cause the so called “painlessness disease”, hereditary sensory and autonomic neuropathy type V (HSAN5) [8,9,10]. Since 2016, it has been widely reported that NGF/TrkA could be a new target to modulate pain [4]. Recently, Peach et al. [11] demonstrated how modulating the NGF response on the TrkA receptor could effectively modulate nociceptive pain. Furthermore, this activity may play a role in modulating nociplastic pain at the spinal level [12], opening up interesting clinical implications for conditions with a nociplastic component, both in acute (e.g., chronic postoperative pain) and chronic pain.

Current clinical applications of TrkA inhibition to treat pain have been well-established [5,13]. Previous studies have identified that the interaction between NGF and TrkA can be impaired by reducing thiolic compounds [14], and recently, in this regard, Govoni and co-workers demonstrated the capability of N-acetylcysteine (NAC) to antagonize NGF activation of TrkA through disulfide bridge interactions [3]. Furthermore, Symon and colleagues, in 2021, evaluated, for the first time, the protective effect of erdosteine on complete Freund’s adjuvant (CFA)-induced arthritis in rats [15]. Moreover, erdosteine demonstrated neuroprotective effects in a rabbit spinal cord injury model by improving neurological outcomes, reducing neuronal damage, and decreasing oxidative stress. These findings suggest its potential as a candidate to be repurposed as a treatment for neuropathic pain [16] and nociceptive pain with a nociplastic component [12] Based on these findings, we have investigated the ability of erdosteine and its primary active metabolite, Met-1, to interact with NGF activation of TrkA in silico and in vitro.

## 2. Results

### 2.1. In Silico Studies

**Figure 1:**
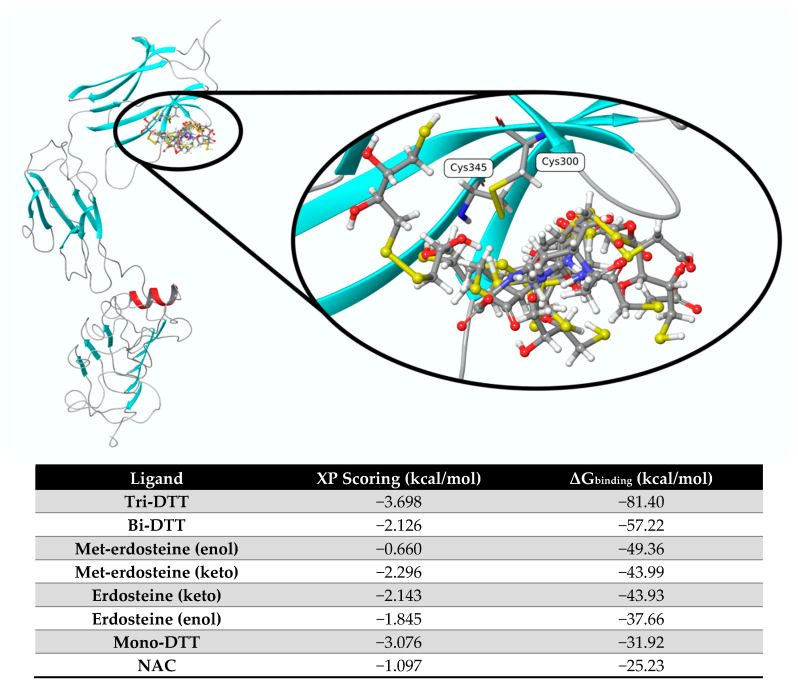
In silico analysis of the interaction between erdosteine/DTT/NAC and TrkA at the disulfide bond between cysteines 300 and 345. DTT: dithiothreitol; NAC: N-acetylcysteine.

We tested the in silico interaction of eight ligands with TrkA, including erdosteine and Met-1, as well as the eno- and keto-tautomers, mono-, bi-, and tri-dithiothreitol (DTT), and NAC as reference substances. Figure 1 shows the pose of each ligand obtained from molecular docking and optimized at the end of the ΔG_binding_ calculation using mm-GBSA. Based on the ΔG values, the most promising ligands are tri-DTT and bi-DTT, which have values of −81.40 kcal/mol and −57.22 kcal/mol, respectively. Erdosteine and Met-1 present a less favorable ΔG_binding_ score. These two ligands can experience keto–enol tautomerism, with the keto form being more dominant than the enol form, and this influences the affinity of the two tautomers. The ligands with the least affinity are mono-DTT and NAC.

**Figure 2:**
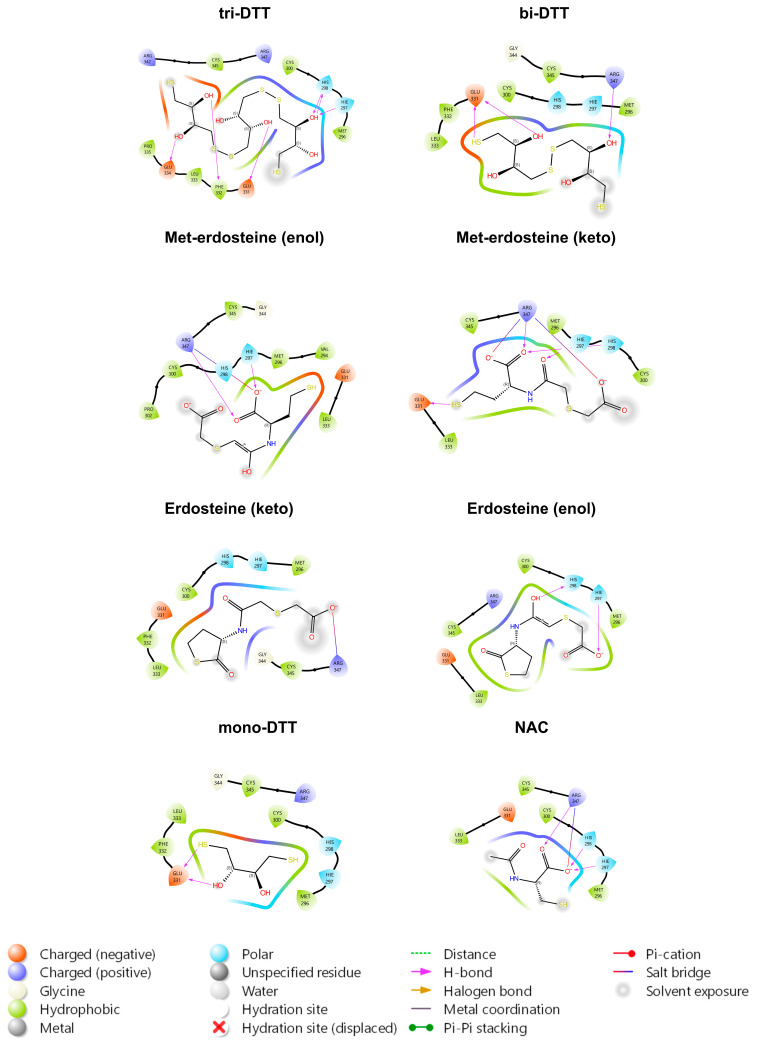

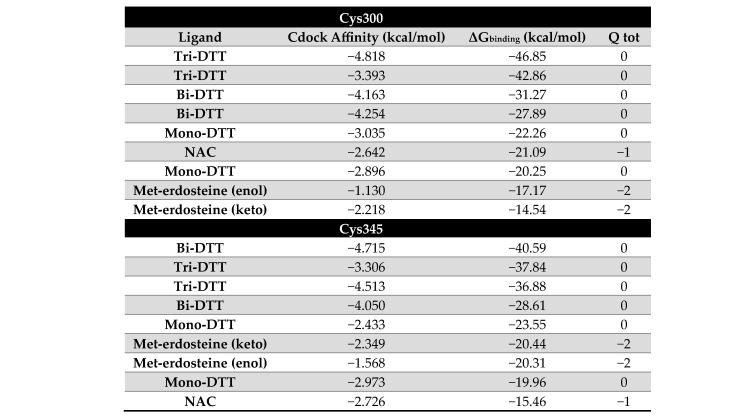
In silico analysis of the interaction between the various ligands and TrkA disulfide bond between cysteines 300 and 345. Representative scheme of a H-bond involving both the Sp–Sp atoms (Sp indicates a sulfur atom belonging to the protein). Representative formation of the Sd–Sp bond between NAC and Cys345 and the protonation of sulfur atom of Cys300. In the associated table, the ability of the various ligands to reduce the disulfide bridge between Cys300 and Cys345 is shown. Erdosteine does not have this property (does not have a free thiolic group) and therefore is not reported. DTT: dithiothreitol; NAC: N-acetylcysteine. The tri/bi/mono-DTT molecules have two reducing ends, both of which have been tested. Such molecules are symmetric; for this reason, the ΔG values calculated for the head and tail ends of the same molecule are very similar. The difference arises from slight variations in the molecule’s orientation caused by the minimization algorithm.

By examining the interactions of all the ligands within the pocket, the primary TrkA residues involved are Glu331, Arg347, His298, and His297. Such residues form hydrogen bonds or salt bridges with the hydroxyl or carbonyl groups of the various ligands. As illustrated in Figure 2, all ligands, except for erdosteine, can reduce the disulfide bridge between Cys300 and Cys345, as assessed through covalent docking simulations and as shown in the table associated with Figure 2. In the case of the two sulfur atoms of erdosteine, one is blocked in the aliphatic side chain, and the other is enclosed in the heterocyclic ring (thioacetone). With these two blocked thiol groups, erdosteine is stable in the dry state or acid media. In vivo (and possibly in the cell culture medium), when passing to a more alkaline environment, the thiolactone ring slowly opens, achieving a transformation to Met-1, thus exposing the free thiol group.

Docking simulations reveal a strong preference for tri-DTT and bi-DTT, whereas methyl-erdosteine shows an affinity similar to NAC, but significantly lower than DTT.

The ligands with higher affinity (DTT) are all neutral at pH 7.0, whereas Met-1 and NAC have a negative charge of −2 and −1, respectively. The binding pocket’s electrostatic potential around the disulfide bridge formed by Cys300 and Cys345 is mainly neutral, which may account for DTT’s higher affinity.

**Figure 3:**
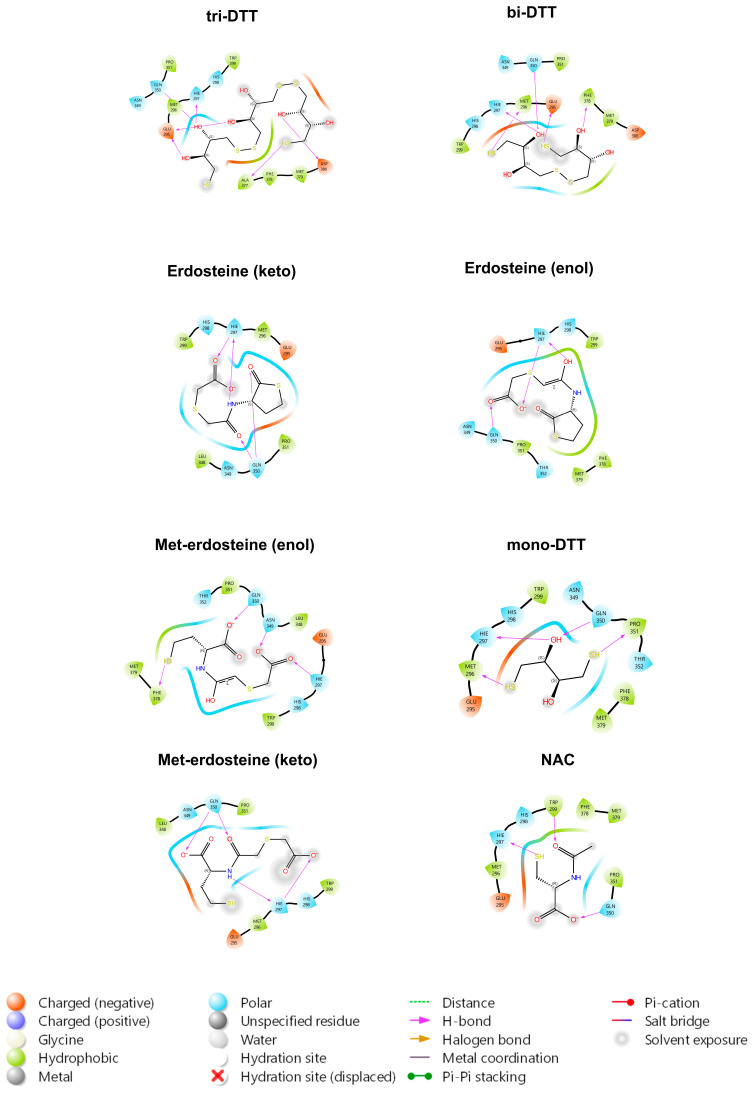

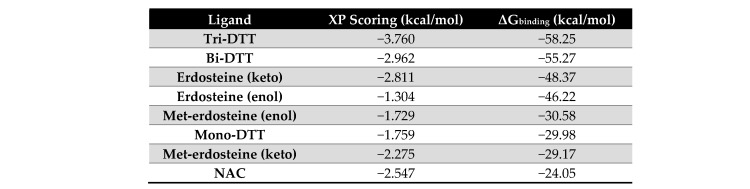
Erdosteine displays a good binding affinity for the region between TrkA and NGF despite not being able to reduce the disulfide bond between Cys 300 and Cys 345. DTT: dithiothreitol; NAC: N-acetylcysteine.

Finally, the ligands were evaluated in the region between TrkA and NGF, with bi-DTT and tri-DTT molecules showing the strongest affinity. Erdosteine also demonstrated good affinity in this region (Figure 3). This finding suggests that the inhibition mechanism may not rely on disulfide bond reduction, since erdosteine cannot carry out this reaction.

### 2.2. In Vitro Studies in Cultured Neuroblastoma Cells

The study was performed in SH-SY5Y cell cultures, analyzing the effect of erdosteine and its active metabolite, Met-1, on NGF-induced TrkA receptor activation. Specifically, TrkA activation was induced using 100 ng/mL NGF for 10 min (min). The choice of NGF concentration and exposure time was based on prior experience with this cell line.

Preliminary experiments confirmed the ability of NGF to elicit a several-fold increase in TrkA autophosphorylation in SH-SY5Y cells. TrkA autophosphorylation is the first step in the cascade of events triggered by NGF binding to the TrkA receptor [17] and thus serves as an index of TrkA activation.

Initially, our experiments were conducted to evaluate the in vitro toxicity of erdosteine and its metabolite up to the concentrations used with other thiol compounds (toxicity might be expected at concentrations around and above 10 millimolar) and to investigate whether the exposure time influenced this parameter. Accordingly, we explored two time points: short (90 min) and long [24 h (hrs)] incubation. In our experience, longer exposure times can sometimes influence the observed response to the test conditions/substances; therefore, it is important to explore an adequate time interval in the initial exploratory protocols.

**Figure 4:**
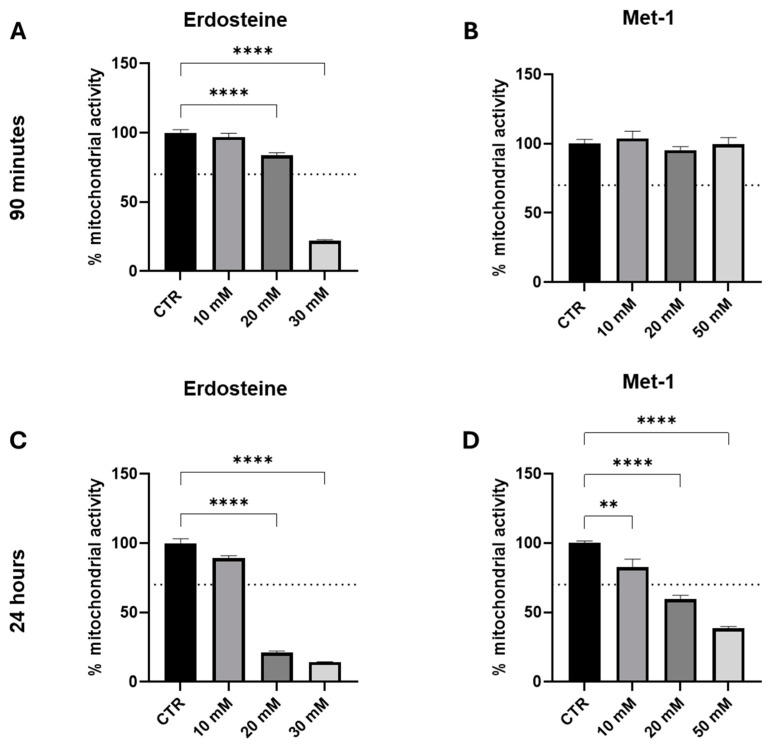
The concentration-dependent effect of erdosteine and Met-1 on the mitochondrial activity of SH-SY5Y cells. The effect of erdosteine (**A**–**C**) and its metabolite Met-1 (**B**–**D**) on cell mitochondrial activity was evaluated, respectively, at 90 min and 24 h. Each value represents the mean ± S.E.M. of independent experiments. Cytotoxic potential is considered present when the cell viability decreases to <70% vs. control group (dotted line). ** *p* ≤ 0.01, **** *p* ≤ 0.0001 vs. control; Dunnett’s multiple comparisons post hoc test; *n* = 6.

As it may be appreciated from the data presented in Figure 4, both erdosteine and Met-1 reduced mitochondrial activity to a greater extent both by increasing the concentration (between 10 and 50 mM) and time of exposure (from 90 min to 24 h). At 90 min, Met-1 was less toxic than erdosteine and did not harm the cells up to the concentration of 50 mM. In contrast, at 24 h both compounds were not tolerated at doses above 10 mM.

**Figure 5:**
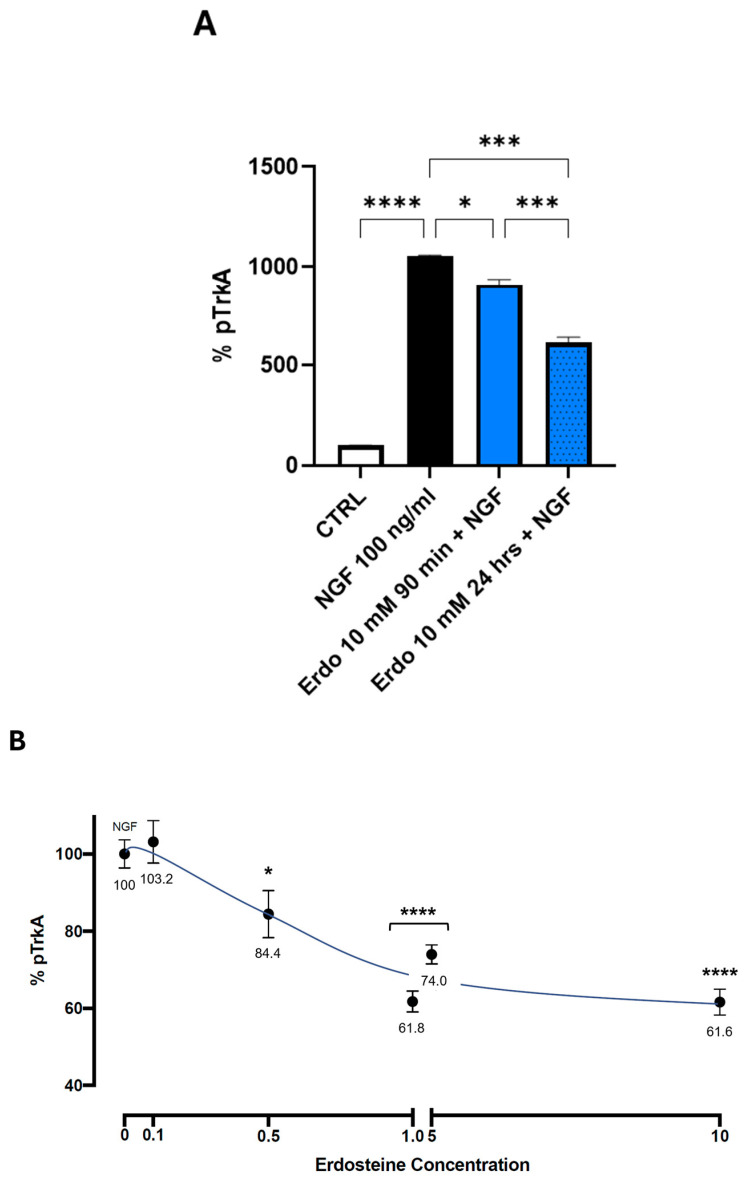
Inhibition of NGF-induced TrkA autophosphorylation by erdosteine. Panel (**A**) reports the effect of exposure to 10 mM erdosteine (Erdo) for 90 min and 24 h. Panel (**B**) reports the effect of various concentrations of erdosteine for 24 h on TrkA autophosphorylation elicited by NGF. The data depicted in (**A**) are reported as % of basal activity, in absence of added substances. The data in (**B**) are expressed as % of the activity in presence of NGF alone (erdosteine concentration of 0, 100% pTrkA) and following the addition of various erdosteine concentrations (from 0.1 to 10 mM) in presence of NGF. Vertical bars represent the standard error of independent experiments (*n* = 3). * *p* ≤ 0.05, *** *p* < 0.001; **** *p* ≤ 0.0001 vs. control. Dunnett’s multiple comparisons post hoc test; *n* = 3. Tukey’s HSD as post hoc test; *n* = 3.

To test the effect of erdosteine or Met-1 on the NGF-induced autophosphorylation of TrkA, we first examined the effect of the maximal tolerable dose of erdosteine (10 mM) after 90 min and 24 h of exposure. Cells were exposed to a medium containing no added substances or 10 mM erdosteine for the times indicated. Then, the medium was changed, and a medium containing NGF 100 ng/mL was added. As shown in Figure 5A, the addition of erdosteine, which per se did not change the basal activity, slightly inhibited (14%), at 90 min, the autophosphorylation elicited by 100 nM NGF. Following 24 h of exposure, the effect was more robust (42% inhibition, *p* < 0.001).

The cells were then exposed for 24 h to the medium containing no added substances or different concentrations of erdosteine (ranging from 0.1 to 10 mM). Then, the medium was changed, and a medium containing NGF 100 ng/mL was added. The inhibition of TrkA activation by NGF in the presence of increasing concentrations of erdosteine, within the interval explored at this time (24 h), was linear between 0.1 and 1.0 mM and then plateaued.

A significant inhibition of NGF-induced TrkA autophosphorylation was observed with 0.5 mM erdosteine.

**Figure 6:**
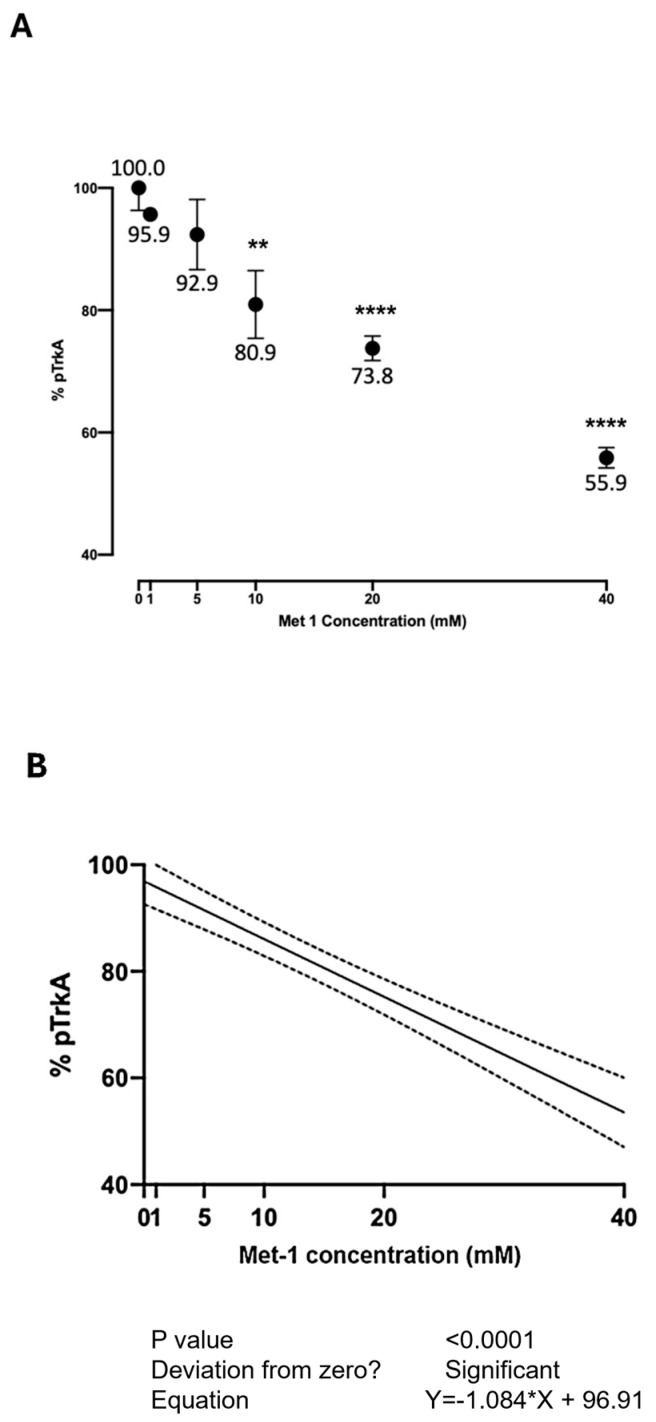
The concentration-dependent effect of Met-1 at 90 min on TrkA autophosphorylation induced by NGF. Panel (**A**) The cells were exposed for 90 min to a medium containing no added substances or Met-1 at various concentrations for 90 min. Then, the medium was changed, and a medium containing NGF 100 ng/mL was added. Data are expressed as % of the activity in response to NGF in absence of added substances (NGF alone, corresponding to 0). Vertical bars represent the standard error of independent samples (*n* = 3 or higher); if not indicated, the bars lie within the symbol dimensions. The inhibition of TrkA activation by NGF in presence of increasing concentrations of Met-1 within the interval explored at this time (90 min) was linear (Panel (**B**)). ** *p* ≤ 0.01; **** *p* < 0.0001 vs. control, Dunnett’s multiple comparisons post hoc test; *n* = 3.

Figure 6 shows the effect of Met-1 at various concentrations on the NGF-induced autophosphorylation of TrkA. Due to the greater cellular toxicity observed with Met-1 following 24 h exposure, the cells were exposed, for 90 min, to the medium containing no added substances or Met-1 at various concentrations for 90 min. Then, the medium was changed, and a medium containing NGF 100 ng/mL was added. The inhibition of TrkA activation by NGF in the presence of increasing concentrations of Met-1, within the interval explored at this time (90 min), was linear (Figure 6B). At the highest tested concentration, Met-1 inhibited TrkA activation by 44.1%.

## 3. Discussion

The in silico results show that erdosteine exhibited good binding affinity to the TrkA receptor (Figure 3), with a binding strength nearly twice that of NAC, but lower than that of tri-DTT and bi-DTT. The higher affinity of tri-DTT and bi-DTT over erdosteine is due to two negatively charged glutamate residues (Glu330 and Glu334) near the binding site, which disfavor the negatively charged erdosteine but not the neutral molecules. The binding occurs within a region of the receptor where NGF binds, promoting a specific rearrangement of the receptor and its autophosphorylation. The primary TrkA residues involved are His298, His297, Glu331, and Arg347, forming hydrogen bonds or salt bridges with the hydroxyl and carbonyl groups of the examined ligands. The metabolite of erdosteine (Met-1), which has a free thiol group and is considered the clinically active metabolite responsible for the mucolytic action of erdosteine, has a lower binding affinity. The keto and enol tautomers of erdosteine and Met-1 have a slight difference in their binding activity for the TrkA receptor. In the case of erdosteine, the keto tautomer is favored, while the opposite is true for Met-1.

NAC had the lowest activity among the tested ligands. Notably, all the ligands, except erdosteine, can reduce the disulfide bridge between Cys300 and Cys345. These results are interesting when considering the varying extents to which these same ligands prevent TrkA activation. While a previous study linked this ability to the property of thiol compounds to reduce disulfide bridges, the present experiments suggest the possibility that solely binding to the pocket may exert the observed inhibition. Indeed, in the case of erdosteine, the compound itself binds to the examined region of interest of TrkA without breaking the Cys300-Cys345 disulfide bridge yet still inhibiting its activation by NGF. However, we cannot exclude the conversion to Met-1 during the 24 h of the in vitro exposure to erdosteine. Over a wide concentration range, the highest inhibition of TrkA activation (44.1%) was observed with 40 mM Met-1 for 90 min. On the other hand, we could not test this concentration after 24 h of exposure because it was too toxic for the SH-SY5Y cells. Compared to our previous experience, both erdosteine and Met-1 were more potent than NAC in inhibiting TrkA activation by NGF. Indeed, concentrations as low as 0.5 mM erdosteine were able to inhibit (by 15.6%) the NGF activation of TrkA, while, in the case of NAC, a 20 mM concentration was needed [3]. It is tempting to speculate that, in vivo, the effect exerted at the TrkA receptor may benefit from the dual action of erdosteine: first directly, and then by breaking the disulfide bridge thanks to the formation of Met-1, owing to its free thiol group capable of breaking the disulfide bond of TrkA Cys300-Cys345. Further experiments should clarify whether this is the sole cysteine bridge involved in the interaction between the studied ligands and TrkA, and whether the p75 receptor for NGF may also play a role. Nevertheless, the investigation of the interaction with the TrkA residues in the binding pocket near the Cys300-Cys345 bridge underscores the importance of this specific region of TrkA in ligand binding.

The data reported in Figure 5 suggest that the maximal inhibition exerted by erdosteine following 24 h of exposure plateaus (around 40% inhibition) between 1 and 5 mM and does not increase further. This is an interesting aspect, suggesting non-complete antagonism due to the interaction with a sequence within the TrkA receptor involving conformational changes favoring activation. The non-complete inhibition of the receptor may be viewed as a positive aspect, since complete antagonism, such as that achieved by NGF monoclonal antibodies in osteoarthritis, is associated with negative consequences due to the disruption of bone remodeling with consequent worsening of osteoarthritis [18]. The partial inhibition by erdosteine, exerted at a non-toxic cellular concentration, may therefore be considered advantageous. Despite the need for in vivo experiments to better define the safety and efficacy of erdosteine in the setting of pain, this drug has been used for up to a year in patients with COPD with a placebo-like side effect profile [19].

As previously discussed, it has been observed that erdosteine exhibits diverse therapeutic actions in respiratory diseases, extending beyond its mucolytic effect. While the pulmonary action of erdosteine in treating pathological conditions such as bronchitis and COPD is the basis of the use of this drug in clinical practice, the present data, together with the observed anti-inflammatory and antioxidant properties described by others, suggest that this substance may also have a therapeutic role in other clinical conditions, including pain [1,2,20].

Clinically, the antioxidant and anti-inflammatory activity of erdosteine has been assessed in COPD patients, where treatment with this drug elicits a significant reduction in the levels of reactive oxygen species (ROS) and of pro-inflammatory cytokines such as IL-8, and 8-isoprostane, a marker of lipid peroxidation in blood, as well as a reduction in exhaled NO [21]. Erdosteine has also been reported to inhibit the release of pro-inflammatory mediators induced by physical exercise in patients with severe COPD and significantly reduced markers of oxidative stress triggered by exertion, further emphasizing the drug’s anti-inflammatory and antioxidant properties [20,22]. However, although these mechanisms of erdosteine have been primarily investigated in relation to pulmonary oxidative stress and inflammation, given that they occur following oral administration, they may also extend to reduce such changes in other conditions associated with the development of acute and chronic pain.

Given the opioid crisis and the adverse effects associated with NSAIDs, the development of effective analgesics for chronic pain conditions is crucial. While several drug treatments, such as antidepressants and anticonvulsants, are available for neuropathic pain, their number needed to treat (NNT) and number needed to harm (NNH) profiles remain suboptimal [23,24].

Recently, supplements have garnered increased attention [25]. However, as the authors acknowledge, limited evidence currently supports their broad clinical use.

Furthermore, the new IASP ICD-11 classification emphasizes “nociplastic pain” [26]. Despite proposed pharmacological interventions, clinical trials specifically investigating their efficacy for this type of pain are scarce [27].

The demonstrated effects of erdosteine position it as a promising candidate to enhance the multimodal approach to pain management, potentially offering a safer alternative in the therapeutic arsenal. Indeed, the available clinical experience of erdosteine in COPD testifies to the good tolerability of the substance at the usual clinical doses [28]. Indeed, clinical studies involving over 2000 patients treated with doses ranging from 600 to 1200 mg for durations of 7 days to 8 months observed no significant differences in side effects (gastrointestinal, cardiovascular, cutaneous, or general reactions) between Erdosteine and placebo [28]. These conditions warrant further investigation with erdosteine using in vivo animal models, and ultimately clinical trials, as has been conducted for other thiol compounds [29,30,31]. In particular, use in characterized in vivo animal models of various painful conditions will help to better identify the clinical applications of this multitargeted molecule.

## 4. Materials and Methods

### 4.1. In Silico Studies

The TrkA structure used is the 2IFG X-ray crystal structure obtained from the Protein Data Bank. The crystallographic structure was processed with Maestro using Protein Preparation Wizard [32] to reconstruct any missing loops or unresolved residues, assign the correct bond order, create disulfide bridges, and generate the correct protonation state of the residues at pH 7.0. The structures of the TrkA–ligand complexes were obtained through a molecular docking simulation conducted with the Glide [33]. A grid box with a size of 20 × 20 × 20 Å was generated around the TrkA region where the NGF co-crystallized moiety shows the minimum average distances. This region is located near the disulfide bridge formed by Cys300 and Cys345 on TrkA. All OH groups present in the docking box are free to move to maximize the formation of hydrogen bond networks. The scoring function used during the simulation is the XP mode of Glide [33]. To enhance result accuracy, the Prime/MM–GBSA method was used to evaluate the binding free energy (ΔG_binding_) of each ligand pose obtained from docking simulation [34]. During this process, a geometry optimization was performed for the entire area within 5 Å of each ligand. Finally, covalent docking simulations were conducted to test the ability of the ligands to reduce the disulfide bridge between Cys300 and Cys345 [35]. All tested ligands were processed to determine the correct protonation state at pH 7.0. All simulations were performed using the OPLS-2005 force field [36]. The ligands tested in the model were erdosteine and its metabolite Met-1, both in the enolic and ketonic form. NAC and mono-, bi-, and tri-DTT were used as reference ligands; other details of the method are provided in Govoni et al., 2024 [3].

### 4.2. Cultured Cells

Human neuroblastoma SH-SY5Y cells were obtained from ATCC (Manassas, VA, USA) and cultured in T75 flasks in a humidified incubator at 37 °C with 5% CO_2_. SH-SY5Y cells were grown in Eagle’s minimum essential medium (EMEM) supplemented with 10% fetal bovine serum, 1% penicillin–streptomycin, L-glutamine (2 mM), non-essential amino acids (1 mM), and sodium pyruvate (1 mM). In MTT experiments, the cells were exposed to 10, 20, and 30 mM erdosteine (Erdo; powder from Edmond Pharma, Paderno Dugnano, Italy); and 10, 20, and 50 mM Metabolite-1 ((±)-N-(2-carboxymethylthioacetyl)homocysteine) (Met-1; powder from Edmond Pharma) for 90 min and 24 h.

For ELISA experiments, the cells were exposed to various concentrations of erdosteine and Met-1 for 90 min and 24 h and then to 100 ng/mL NGF (rh beta-NGF; SRP3015, Sigma-Aldrich, St. Louis, MO, USA) for 10 min. The concentration and the time chosen for NGF were the best combination as derived from preliminary experiments using concentrations of NGF ranging from 0.5 to 1000 ng/mL and times from 5 to 60 min, eliciting TrkA autophosphorylation response ranging from 2.5 to several folds the basal rate. In the preliminary experiments, NAC 20 mM was used as reference compound derived from our previous experience. All the experiments were performed under a laminar flow hood.

### 4.3. MTT Assay

Mitochondrial enzymatic activity was estimated by MTT [3-(4,5-dimethylthiazol-2-yl)-2,5-diphenyltetrazolium bromide] assay (Sigma-Aldrich). A cell suspension of 2 × 10^4^ cells/mL SH-SY5Y cell line was seeded into 96-well plates. Following each treatment of 90 min and 24 h, 50 μL of MTT (concentration equal to 2.5 mg/mL) was added to each well. After incubation at 37 °C for 3 h, the purple formazan crystals were formed. The formed crystals were solubilized in dimethylsulfoxide (DMSO; Sigma-Aldrich). Specifically, after removing the MTT from the wells, 100 μL of DMSO was added to lyse the cellular and mitochondrial membranes and solubilize the formazan crystals. After 10 min, the absorbance values were measured at 595 nm using a Synergy HT microplate reader (BioTek Instruments, Winooski, VT, USA) and the results expressed as % with respect to control.

### 4.4. ELISA Assay

The phospho-TrkA (Tyr674/675) protein levels in SH-SY5Y cells were estimated with a specific ELISA kit (Cell Signaling, Danvers, MA, USA), according to the manufacturer’s instructions. PathScan^®^ Phospho-TrkA (Tyr674/675) Sandwich ELISA Kit is a solid phase sandwich enzyme-linked immunosorbent assay that detects transfected levels of phospho-TrkA (Tyr674/675) protein. After incubation with the cell lysates, both phospho- and nonphospho-TrkA proteins are captured by the coated antibody. Following extensive washing to remove any unbound antibody/reagent, phospho-TrkA (Tyr674/675) antibody is added to detect phospho-TrkA protein. Anti-rabbit IgG, HRP-linked antibody is then used to recognize the bound detection antibody. The HRP substrate, TMB (3,3′,5,5′-tetramethylbenzidine), is added to develop the color. The color development was stopped and the intensity of the color measured at 450 nm using a Synergy HT microplate reader (BioTek Instruments). The magnitude of the optical density for this developed color is proportional to the quantity of phospho-TrkA (Tyr674/675) protein. Note that, according to the producer, all the antibodies used in the kit were custom formulations specific for the kit.

### 4.5. Statistical Analysis

Mean and standard deviation were calculated for each variable. To compare the various in vitro treatments, post hoc pairwise comparisons were performed using Dunnett’s multiple comparisons test following one-way ANOVA, considering *p* < 0.05 as the threshold of statistical significance. In the case of Figure 5A we used the Tukey’s HSD test which was more appropriate due to the additional between groups comparison. This has been added in the Figure 5 legend and in the methods. GraphPad Prism software 9.0 was used for statistical analysis.

## Data Availability

All data generated or analyzed during this study are included in this article.
